# CYRI/ Fam49 Proteins Represent a New Class of Rac1 Interactors

**DOI:** 10.1080/19420889.2019.1643665

**Published:** 2019-07-23

**Authors:** Jamie A. Whitelaw, Sergio Lilla, Nikki R. Paul, Loic Fort, Sara Zanivan, Laura M. Machesky

**Affiliations:** a CRUK Beatson Institute, University of Glasgow, Glasgow, UK; b Department of Cell and Developmental Biology, Vanderbilt University School of Medicine, Nashville, TN, USA; c Institute of Cancer Sciences, University of Glasgow, Glasgow, UK

**Keywords:** GTPase signaling, Rac1, cell migration, actin, cancer invasion

## Abstract

Fam49 proteins, now referred to as CYRI (CYFIP-related Rac Interactor), are evolutionarily conserved across many phyla. Their closest relative by amino acid sequence is CYFIP, as both proteins contain a domain of unknown function DUF1394. We recently showed that CYRI and the DUF1394 can mediate binding to Rac1 and evidence is building to suggest that CYRI plays important roles in cell migration, chemotaxis and pathogen entry into cells. Here we discuss how CYRI proteins fit into the current framework of the control of actin dynamics by positive and negative feedback loops containing Rac1, the Scar/WAVE Complex, the Arp2/3 Complex and branched actin. We also provide data regarding the interaction between Rac1 and CYRI in an unbiassed mass spectrometry screen for interactors of an active mutant of Rac1.

## Regulation of Arp2/3 driven actin assembly at the lamellipodium

**Figure 1.**
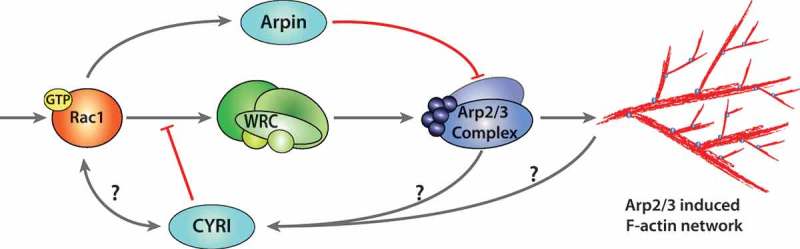
Negative regulation of actin polymerisation at the lamellipodium. Actin polymerisation at the lamellipodium is activated through the Rac1-WRC-Arp2/3 cascade. Extracellular stimuli trigger GTP loading of the small GTPase Rac1 and plasma membrane association. Activated Rac1 interacts with WRC through CYFIP1 at either or both the A- and D-sites, releasing and presenting the VCA-domain of WAVE to the Arp2/3 complex. Arp2/3 triggers nucleation of branched F-actin at the leading edge, generating a lamellipodium. Negative regulation of Arp2/3 can occur through the interaction with Arpin, which is activated by Rac1. CYRI proteins compete with the WRC for active Rac1, thereby inhibiting Arp2/3 complex activation. The mechanism of activation of CYRI proteins to constrain protrusions is still unknown. The activation cascade follows grey arrows, while inhibitors are shown in red terminal lines.

Orchestrated directional cell migration is fundamental for multiple aspects of life; from development, wound healing and immune responses to pathogens. At the core of cell migration is the cells ability to rearrange its actin cytoskeleton, which is driven predominantly by the Arp2/3 complex. During this process, extracellular cues trigger intracellular signalling cascades resulting in the activation of the small GTPases Rac1 and Cdc42 at the leading-edge plasma membrane. In the case of Rac1, these signals facilitate the interactions and subsequent activation of the WAVE-regulatory complex (WRC). The WRC is a pentameric complex composed of 5 members; CYFIP1/2 (Sra/PIR121), NckAP1/2 (Hem-1/2), WAVE1-3 (Scar1-3), HSPC300 (Brick1) and Abi1-3 [1]. Interaction with Rac1 causes conformational changes in the complex, releasing the autoinhibited C-terminal Arp2/3 binding domain of WAVE, which in turn activates the Arp2/3 complex [2–4] (Figure 1).

A recent study described two possible binding sites for Rac1 on the WRC. These sites are referred to as A and D- sites, with the D-site being first described by Chen et al., 2017. The D-site has been described by cryo-electron microscopy and is located within the more C-terminal fragile X mental retardation protein (FMRP) region of CYFIP [3,5]. Chen and colleagues found that Rac1 binds to the D-site of CYFIP with a greater affinity than the A-site alone. However, Rac1 binding to both regions on CYFIP is needed to cause full activation of WRC [3]. The A- and D-sites were confirmed to interact with active Rac1 within both mammalian and *Dictyostelium* cells [5]. Moreover, while the A-site was required for WRC activation, the D-site was surprisingly dispensable for WRC activation but necessary for proper lamellipodial formation and functions, suggesting that both sites differentially contribute to the activation and stability of the WRC for lamellipodium dynamics [5].

The downstream activation of the Arp2/3 complex triggers actin polymerisation at the leading edge and also the formation of branched F-actin [2,6]. This generates broad, flat membrane protrusions termed lamellipodia, which are characteristic of cells migrating on flat rigid substrates. To respond to directional cues rapidly, cells must have a fine-tuned balance between protrusion and retraction to permit steering and chages in direction. Through decades of research, a pathway linking the Rho family GTPase Rac1, the WRC and the Arp2/3 complex has been established for lamellipodia generation and migration [7,8] and reviewed in [9]. While the mechanisms required to activate actin polymerisation at the leading edge are still extensively studied, how negative regulation allows plasticity and retraction is less well understood. Gautreau and Krause have highlighted the known positive and negative feedback loops controlling branched actin assembly [9]. Negative regulators of Arp2/3 complex have been described, such as PICK1, Gadkin and Arpin. However, only Arpin inhibits the Arp2/3 complex at the lamellipodium, while Gadkin and PICK1 play a role at surface endosome and clathrin-coated pits, respectively [10–12]. Interestingly, some activators and inhibitors of the Arp2/3 complex co-localise at membrane sub-compartments of the cell, such as Arpin and WAVE at the lamellipodium, Gadkin and WASH at endosomes and PICK1 and WASP at clathrin-coated pits [13]. Gadkin and PICK1 both contain an acidic motif resembling the tail of WASP-family proteins, but instead of activating Arp2/3, they seem to be antagonistic. Arpin was identified through a bioinformatics search for COOH-terminal acidic A-motifs found in nucleation promoting factors (NPFs). Arpin localises to the lamellipodia and acts downstream of Rac1 to competitively inhibit binding of WAVE to Arp2/3 by exposing the A-motif to Arp2/3 and keeping it in an inactive confirmation [10,13]. This causes a reduction in lamellipodia protrusion lifetime and directional persistence for migration [10], although it is dispensable for chemotaxis [14].

## A role for CYRI in membrane protrusions and cell migration

All negative regulators of this pathway identified prior to CYRI are inhibitors of the Arp2/3 complex, raising the question: are there novel regulators at the level of the WRC? Using a tagged version of the *Dictyostelium discoideum* WRC subunit Nap1 (NckAP1 in human), CYRI was identified by mass spectrometry through a reversible formaldehyde cross-linked, GFP-trap pulldown [15]. CYRI proteins are highly conserved through evolution, where mammals have two isoforms, CYRI-A and CYRI-B. The protein sequence of both CYRI isoforms comprise an N-terminal predicted alpha helix with a concensus myristoylation site, followed by a domain of unknown function 1394 (DUF1394) domain, which has sequence homology to the WRC subunit CYFIP1. The Rac1 binding A-site in CYFIP1 resides in the DUF1394, interacting specifically with active forms of Rac1 but not Cdc42 or RhoA [15]. Depletion of CYRI-B and subsequent optogenetic activation of Rac1 promoted broader and less dynamic lamellipodia, similar to the fried egg phenotype of cells with constitutively active Rac1 as previously described [16]. Conversely, overexpression of CYRI-B resulted in cells shrinking in area and having unproductive protrusions. Localisation of CYRI-B to the leading edge of cells could only be observed in fixed assays with a FLAG-tagged construct, which inhibited many cells from forming lamellipodia. It is possible that larger C- or N-terminal tags such as GFP inhibit the regulation of CYRI-B by interfering with N-terminal myristoylation [15]. CYRI-B functions to restrict protrusion lifetime and promote pseudopod splitting by mimicking the Rac1-CYFIP interaction. It is therefore required to regulate polarity and plasticity of protrusions for effective migration and chemotaxis [15].

Hans Meinhardt proposed that a minimal mathematical model of cell migration could be made using a global inhibitor, an activator and a local inhibitor [17]. The local inhibitor needed to be smaller than the activator (to diffuse more rapidly) and to be recruited by the same signal as the activator. We postulate that WRC and active Rac1 are the activators, while CYRI-B plays the role of the local inhibitor by restricting Rac1’s activity at the cell membrane [15] (Figure 1). While this model is a good starting point to understand lamellipodial feedback loops, we are still left with further questions surrounding the mechanisms of activation/inactivation of CYRI.

### Rac1-mediated pathways in cancer

Migration of tumour and associated cells is a key part of cancer cell metastasis, which relies on the dynamic changes of the actin cytoskeleton. Some studies have correlated expression of components of the actin machinery with cancer progression, but the importance of this is not clear beyond a general correlation with increased actin dynamics when cells are more mesenchymal and thus thought to be more agrressive [13,18–20]. Reduced expression of *Cyfip1* or *NckAP1* has been linked to increased invasiveness, indicating that they have the potential to act as tumour invasion suppressors [21,22]. *In vitro*, silencing of either *Cyfip1* or *NckAP1* in cancer cells migrating in 3D caused cells to become more invasive due to increased reliance on an N-Wasp driven Arp2/3-mediated migration [22]. However, in breast cancer cells, silencing of NckAP1 or disrupting the Arp2/3 complex reduced invasion [23]. Moreover, following subcutaneous implant, there was no difference in primary tumour volume, but a reduction in the number of lung metastases of MDA-MB-231 cells [23], indicating potential involvement of WRC in metastatic dissemination.

Rac1 mediates activation of the WRC and resulting actin polymerisation and can be hyperactivated in cancers. An activating mutant of Rac1 containing a proline to serine change, Rac1^P29S^, was found in 9% of all sun-exposed melanomas, placing it as one of the top 10 mutated genes (behind BRAF, NRAS, TP53, PTEN and others) [24]. Rac1^P29S^ shows a high GDP/GTP exchange and thus is hyperactive. Similar to the constitutively active Rac1^Q61L^, the Rac1^P29S^ mutation causes an increase in cell migration, membrane ruffles and proliferation [25,26].

Before it was known to be a Rac1 interacting protein, CYRI-B was highlighted as being highly expressed in pancreatic cancer [27]. CYRI-B knockdown PDAC cells were injected in the tail vein of immunocompromised mice, resulting in increased lung colonisation and enhanced growth compared with control cells [27]. This may be due to enhanced Rac1 activity in CYRI-B knockout cells [15]. Rac1 has previously been implicated in pancreatic cancer progression [28] and is thought to regulate actin localisation and dynamics during dysplastic changes in precancerous regions of pancreas as well [29]. In summary, disrupting the regulation of Rac1 signaling and actin dynamics may drive the invasiveness and spread of various cancers and the role of CYRI in this process warrants further study.

## Beyond actin dynamics

Chattaragada et al., found that silencing of CYRI-B in PDAC cells lead to an increase in mitochondrial O_2_^−^ levels, resulting in stress due to reactive oxidative species (ROS). This rise in ROS levels was linked to enhanced tumour cell proliferation and invasiveness [27]. In neutrophils, activation of Rac1 is well known to enhance superoxide production via direct binding to the phagocyte oxidase complex PHOX [30,31]. However, in other cell types, NOX plays the role of PHOX and its regulation is much less well understood [31,32]. It is possible that higher levels of Rac1 activity in CYRI knockout cells leads to activation of NOX and thus induces greater superoxide production observed by Chattaragada and colleagues [27]. Alternatively, Rac1 has been localised to the mitochondrial membrane and is implicated in maintenance of superoxide production homeostasis through its interaction with BCL-2 [33]. Inhibiting the BCL-2-Rac1 interaction in lymphoma cells, decreased the mitochondrial O_2_^−^ levels and lead to the triggering of apoptosis [33]. Furthermore, Chattaragada et al. reported mitochondrial localisation of CYRI-B [27]; although we could not confirm this observation either in mammalian cells or *Dictyostelium* thus far [15]. It remains for the future to discover the mechanism by which CYRI may impact on superoxide production and whether this is related to Rac1 control of NOX or mitochondrial ROS production.

Additionally, Yuki et al. [34] report that CYRI-B restricts Salmonella infection in mice, as they identified CYRI in a screen for novel genes that when mutated affected susceptibility to infection. This suggests a possible immune function for CYRI-B, possibly at the level of bacterial uptake, which requires engagement of the actin cytoskeletal machinery and Rac1 activation [34]. They further show in this study that CYRI-B can be destroyed by ubiquitination downstream of Rac1 activation by the Salmonella effector SopE, suggesting interesting possibilities for regulation of CYRI-B in cells [34].

## Proteins that bind specifically to active Rac1

**Figure 2.**
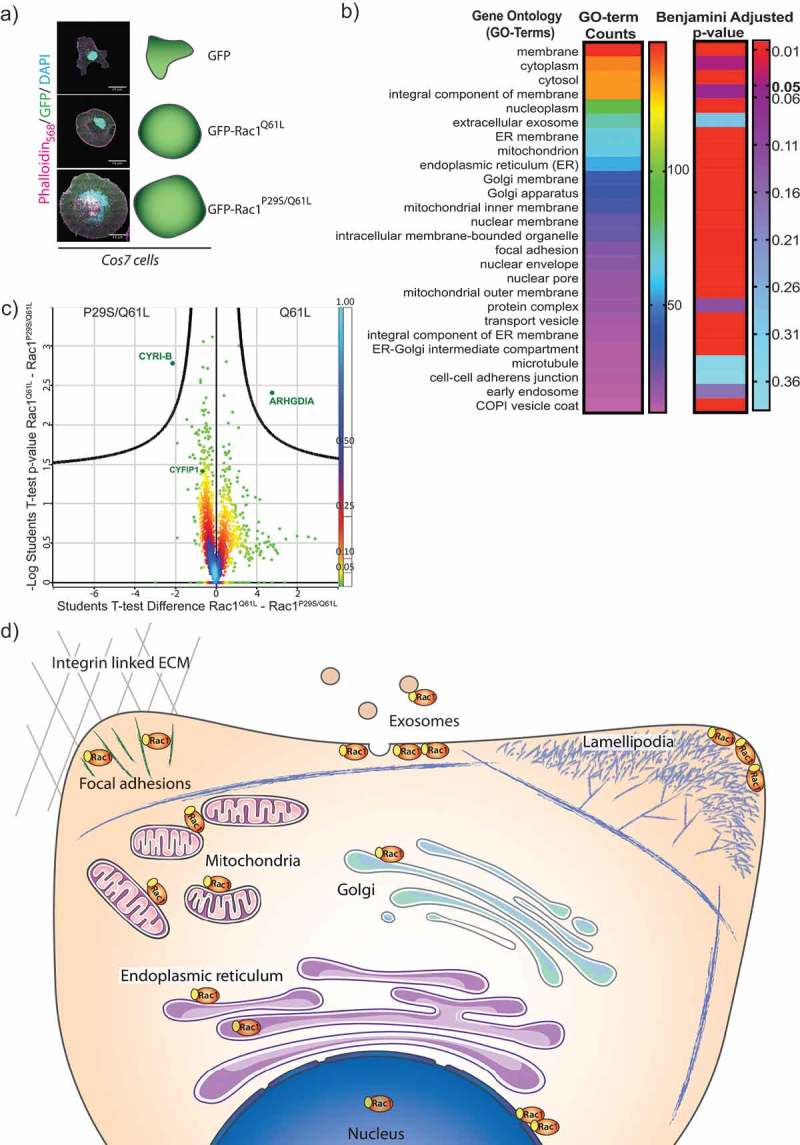
Screen for active Rac1 interacting proteins. A) Cos7 cells transfected with GFP, GFP-Rac1^Q61L^ or GFP-Rac1^P29S/Q61L^ for immunoprecipitation with GFP-pulldown, Scale bar 25 μm. B) GO-term analysis (*Homo sapiens*) to sort significantly enriched proteins that bound to both forms of active Rac1 compared to GFP into categories based on number of hits. Adjusted p-values using the Benjamini False discovery rate (FDR) scoring shows significance of those hits using a rainbow scale with p-value 0.05 highlighted. C) Volcano plot illustrating results from t-test applied on protein intensity differences between the two Rac-1 mutants (GFP-Rac1^Q61L^ and GFP-Rac1^P29S/Q61L^) measured in liquid chromatography–tandem mass spectrometry experiments. The colour coding is based on density of the data points, the scale is indicated on the right, and the curved line shows the 5% FDR. D) Schematic representation of a cell and the important regions of Rac1 activity based on the GO-term locations.

CYRI-B interacts with active forms of Rac1, such as Rac1^P29S^, Rac1^Q61L^ [15,34] and interestingly appears to show increased affinity to double mutant Rac1^P29S/Q61L^ [15]. Rac1^P29S/Q61L^ was first described by Chen and colleagues [3] but is not a naturally occurring mutation (to our knowledge). It is unclear how this double mutation affects Rac1 structurally, including GTP binding or hydrolysis. We speculated that this mutant might have higher affinity for DUF1394 domains, since we and Chen both found enhanced binding to CYRI and CYFIP. To test whether any other motifs or proteins were preferred by Rac1^P29S/Q61L^ over the more conventional single mutation Rac1^Q61L^, we transfected cells with either GFP, GFP-Rac1^Q61L^ or GFP-Rac1^P29S/Q61L^ (Figure 2A) and used GFP-trap and mass spectrometry to compare binding partners. We retrieved many known Rac1-interacting proteins that were grouped into Gene Ontology (GO)-terms (Figure 2B). Additionally many of the target genes were in clusters around membrane-bound organelles such as the nucleus and mitochondria (Figure 2B and see also Supplementary Table 1). Details of proteins interacting with either mutant active Rac1 protein can be found in Supplementary Table 1 and the data are available via ProteomeXchange with identifier PXD013941.

To our surprise, CYRI-B was the only protein retrieved with a statistically significant ratio that interacted preferentially with Rac1^P29S/Q61L^ compared to Rac1^Q61L^ (Figure 2C). On the other hand, ARHGDIA (also known as RhoGDI1) showed significantly less binding to the double mutant Rac1^P29S/Q61L^ than Rac1^Q61L^. RhoGDI is the major regulator of GDP-bound inactive Rho GTPases Rac1^Q61L^ [35] (Figure 2C). This could be a clue as to why the double mutant Rac is more available for binding to CYRI-B, but does not explain why we did not retrieve CYFIP1 in this experiment nor why CYRI-B and presumably the DUF1394 uniquely prefers this mutant form of Rac1. Notably, the NonO proteins, found to control nuclear actin and transcription [36], also bound preferentially to Rac1^Q61L^. Finally, we highlight proteins from our screen that interact with Rac1 ^Q61L^ and Rac1 ^P29S/Q61L^ at specific regions within the cell (Figure 2D). This follows recent literature showing diverse functions for Rac1 activity throughout the cell [37].

In summary, CYRI proteins are novel regulators of lamellipodia dynamics through their interaction with Rac1 at the DUF1394 domain. CYRI proteins negatively regulate the activity of the WRC and suppress lamellipodia spread and dynamics of protrusions. Cells need CYRI proteins to maintain plasticity and to allow leading edge actin dynamics to be able to rapidly respond to changing environments. While CYRI’s role in cells is starting to become clear, much remains to be learned about the roles of CYRI proteins in healthy tissues, organisms and in cancer.

## Methods

### Transfection and protein isolation

Cos-7 (chlorocebus sabeus fibroblast) cells grown at 37°C and 5% CO2, were transfected with either GFP, GFP-Rac1^Q61L^ (human) or GFP-Rac1^P29S/Q61L^ using Lipofectamine2000 (Invitrogen) following the manufacturer’s protocol. The transfected cells were left overnight at 37°C, 5% CO_2_ and replated the next day on laminin-coated dishes and incubated overnight.

The plates were washed twice in ice-cold PBS and lysed in GFP-trap buffer (25mM Tris HCl, pH7.5, 100mM NaCl, 5mM MgCl_2_, 0.5% NP-40, containing protease and phosphatase inhibitors). Around 1.5 mg of lysates were incubated with GFP-Trap_A beads (ChromoTek) for 2 hours at 4°C following the manufacturer’s protocol. Beads were washed three times with Wash buffer (25mM Tris HCl, pH7.5, 100mM NaCl, 5mM MgCl_2_, containing protease and phosphatase inhibitors). The beads were stored at −20°C in a solution of 100 mM ammonium bicarbonate + 2 M UREA Prior to digestion.

### On-beads proteolytic digestion

Immunoprecipitations were performed in triplicate, and purified proteins were digested on beads using the method described in [38].

### Liquid chromatography–mass spectrometry

Tryptic digests were obtained and separated by nanoscale C18 reverse-phase liquid chromatography using an EASY-nLC 1200 (Thermo Fisher Scientific) coupled to an Orbitrap Q-Exactive HF mass spectrometer (Thermo Fisher Scientific). The eluting peptide solutions were introduced into the mass spectrometer via a nanoelectrospray ion source (Sonation). The mass spectrometer was operated in positive ion mode and used in data-dependent acquisition.

### Data analysis

The MS Raw data were processed with MaxQuant software [39] and searched with Andromeda search engine [40], querying both UniProt [41] *Chlorocebus sabaeus* (13/03/2017; 19,228 entries) and *Homo sapiens* (09/07/2016; 92,939 entries) plus the sequence of GFP-Rac1 (Q61→L) and GFP-Rac1 (P29→S and Q61→L) construct used in the experiment. Combined databases were searched assuming the digestion enzyme trypsin allowing for two miscleavages. Methionine oxidation and N-terminal acetylation were specified as variable modifications, and Cysteine carbamidomethylation was specified as fixed modifications. The peptide, protein and site false discovery rate (FDR) was set to 1%. The common reverse and contaminant hits (as defined in MaxQuant output) were removed. Only protein groups identified with at least one uniquely assigned peptide were used for the analysis. For label-free quantification, proteins were quantified according to the label-free quantification algorithm available in MaxQuant [42]. Accurate proteome-wide label-free quantification by delayed normalization and maximal peptide ratio extraction, termed MaxLFQ. Three independent replicates were generated per condition (GFP control and two RAC-1 mutants used in the immunoprecipitations), and significantly enriched proteins were selected using a t-test based analysis (5% False Discovery Rate).

## Go-term analysis

Genes derived from mass spectrometry results were scored for significance for both active Rac1 screens and absent from GFP controls were then uploaded to DAVID (Database for Annotation, Visualisation and Integrated Discovery) v6.8 for GO-term themed searches following the protocol described in [43]. We used *Homo Sapiens* as the species as this closely relates to *Chlorocebus sabaeus* but has more GO-terms available.
